# An ortho­rhom­bic polymorph of isavuconazole

**DOI:** 10.1107/S2056989025008886

**Published:** 2025-10-17

**Authors:** Anna Ben, Lilianna Chęcińska

**Affiliations:** a University of Lodz Doctoral School of Exact and Natural Sciences Narutowicza 68 90-136 Łódź Poland; b University of Lodz Faculty of Chemistry Pomorska 163/165 90-236 Łódź Poland; Katholieke Universiteit Leuven, Belgium

**Keywords:** isavuconazole, crystal structure, polymorph, energy frameworks

## Abstract

The title compound C_22_H_17_F_2_N_5_OS (systematic name: 4-{2-[(2*R*,3*R*)-3-(2,5-di­fluoro­phen­yl)-3-hy­droxy-4-(1,2,4-triazol-1-yl)butan-2-yl]-1,3-thia­zol-4-yl}benzo­nitrile), represents a new ortho­rhom­bic polymorph of isavuconazole. The two stereogenic centers adopt the *R*,*R* configuration. In the crystal structure of the ortho­rhom­bic form, a mono-periodic chain motif is formed by a strong O—H⋯N hydrogen bond, while three additional C—H⋯N inter­actions propagate these chains into a tri-periodic supra­molecular network. A comparison with the previously reported monoclinic polymorph [Voronin *et al.* (2021 ▸). *CrystEngComm***23**, 8513] is provided, supported by Hirshfeld surface and energy framework analyses.

## Abstract 2

The crystal structure of a new ortho­rhom­bic polymorph of pure isavuconazole has been determined and and is compared with the known monoclinic form.

## 1. Chemical context

Heterocyclic compounds have long attracted considerable attention due to their importance as structural inter­mediates in many biologically active substances (Raman *et al.*, 2025 ▸). For over a century, they have been one of the key areas of research in organic chemistry. Nitro­gen-containing heterocycles, in particular, exhibit a wide range of applications, from pharmaceuticals and agriculture, through materials science and coordination chemistry, to the dye and pigment industry (Salma *et al.*, 2024 ▸). Among these, triazole derivatives have drawn significant inter­est in recent decades due to their diverse chemical and biological activities, including anti­fungal (Li *et al.*, 2019 ▸), anti­cancer (Slaihim *et al.*, 2019 ▸), and anti­bacterial properties (Hussain *et al.*, 2019 ▸).
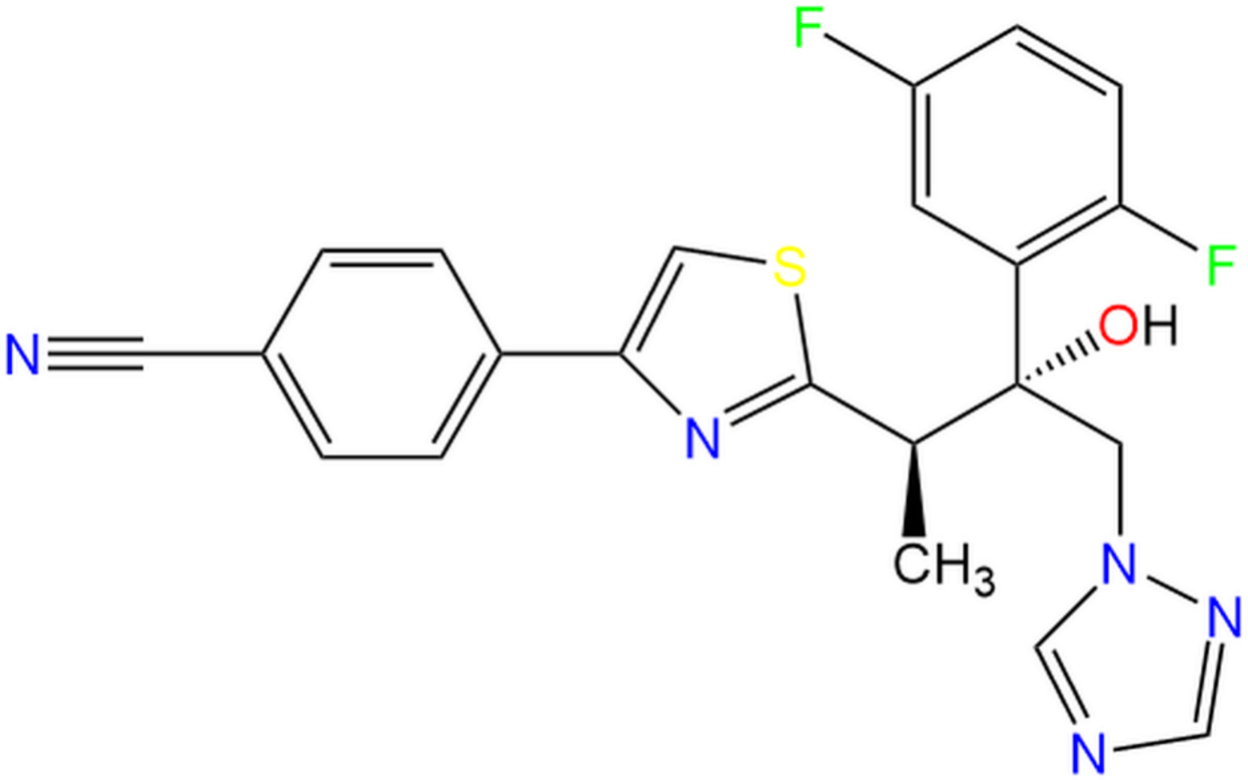


Isavuconazole is a novel and promising broad-spectrum triazole used to treat invasive fungal infections in humans (Shirley & Scott, 2016 ▸). This drug is available in both intra­venous and oral formulations (Lewis II *et al.*, 2022 ▸) and demonstrates activity against yeasts, molds, and dimorphic fungi. Moreover, it has been approved for the treatment of invasive aspergillosis and mucormycosis (Miceli & Kauffman, 2015 ▸).

Only four crystal structures of isavuconazole have been reported to date: the pure form, a monohydrate, and two salts (Voronin *et al.*, 2021 ▸). In this article a crystal structure of a new ortho­rhom­bic form of pure isavuconazole (ISV-ortho) is reported, and compared with its monoclinic form (ISV-mono) (Voronin *et al.*, 2021 ▸).

## 2. Structural commentary

**Figure 1:**
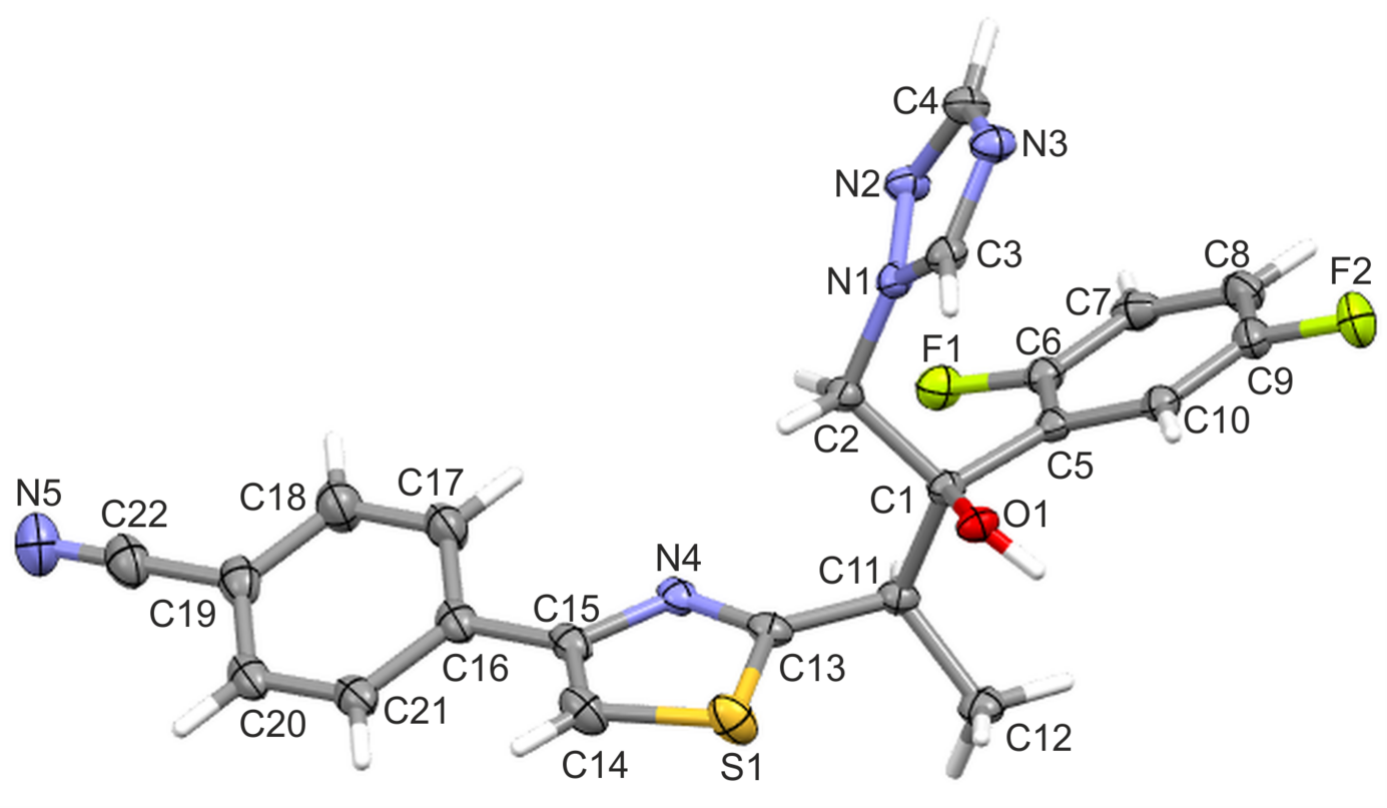
The mol­ecular structure of ISV-ortho with the atom-numbering scheme. Displacement ellipsoids are drawn at the 50% probability level and H atoms are shown as small spheres of arbitrary radii.

The title compound is ortho­rhom­bic, crystallizing in space group *P*2_1_2_1_2_1_. The mol­ecule (Fig. 1 ▸) consists of four rings (1,2,4-triazole, 2,5-di­fluoro­phenyl, 1,3-thia­zole and benzo­nitrile) and a hydroxyl group connected to each other by a flexible chain. In both polymorphs of isavuconazole, ISV-ortho and ISV-mono, the stereogenic centers at C1 and C11 adopt an *R*,*R* configuration.

**Figure 2:**
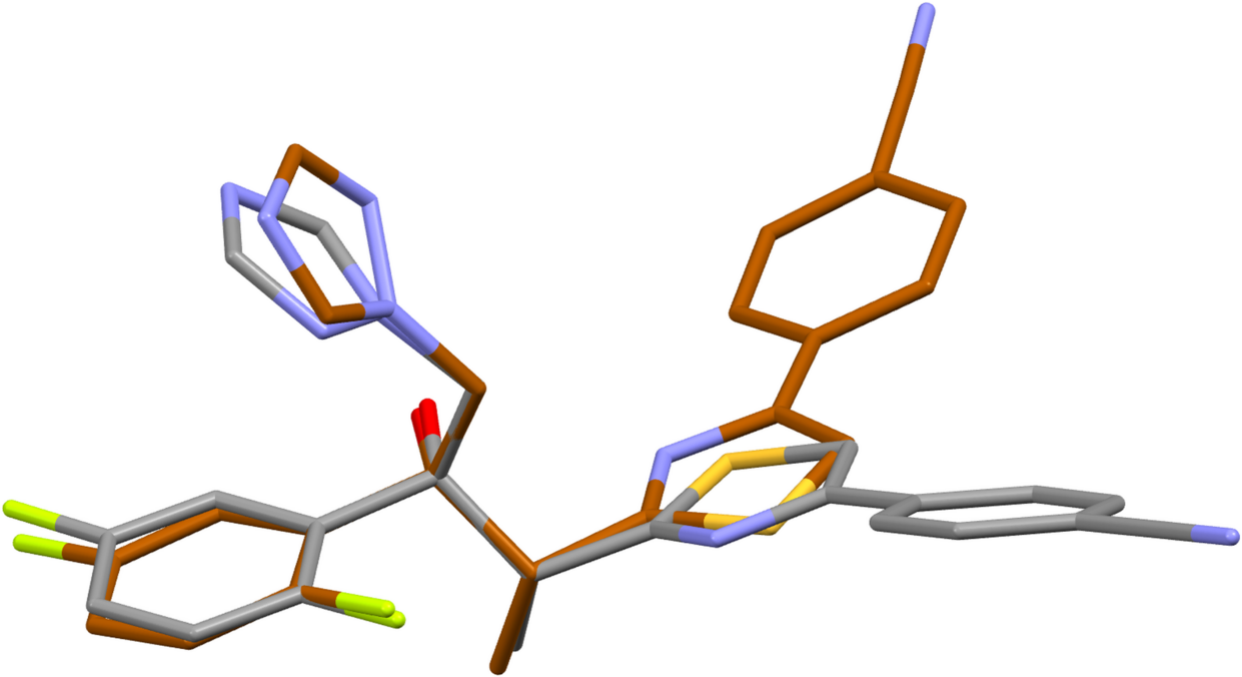
Overlay of the title mol­ecule (ISV-ortho) and the monoclinic polymorph (ISV-mono) with carbon atoms shown in brown.

The superposition of the two polymorphs reveals clear conformational differences (Fig. 2 ▸). The main distinction lies in the orientation of the 4-(thia­zol-4-yl)benzo­nitrile fragment, which is rotated by approximately 180°. Differences are also evident in the torsion angles: in the ortho­rhom­bic form, the torsion angles are −81.6 (2)° (C1—C11—C13—S1) and −37.3 (2)° (C2—C1—C11—C13), whereas in the monoclinic form the corresponding torsion angles are −118.4 (1) and −58.5 (2)°, respectively. Additionally, the dihedral angle between the triazole ring and the 2,5-di­fluoro­phenyl ring is 40.14 (8)° in ISV-ortho and 67.6 (1)° in ISV-mono.

## 3. Supra­molecular features

The monoclinic polymorph of isavuconazole features only C—H⋯*X* (*X* =N/O/F) hydrogen bonds along with inter­actions involving π-electrons, such as C—H⋯π and aromatic π–π stacking inter­actions (Voronin *et al.*, 2021 ▸). A quantum topology analysis revealed that the strongest individual inter­molecular inter­action in pure ISV-mono does not exceed 11 kJ mol^−1^. This was taken to indicate a tendency of the API to exhibit amorphization or polymorphism due to the absence of persistent packing motifs (Voronin *et al.*, 2016 ▸, 2021 ▸).

In this study, we compare the supra­molecular architectures of both polymorphs of ISV based on the analysis of inter­actions energies using the pairwise model implemented in *CrystalExplorer* (Spackman *et al.*, 2021 ▸).

**Figure 3:**
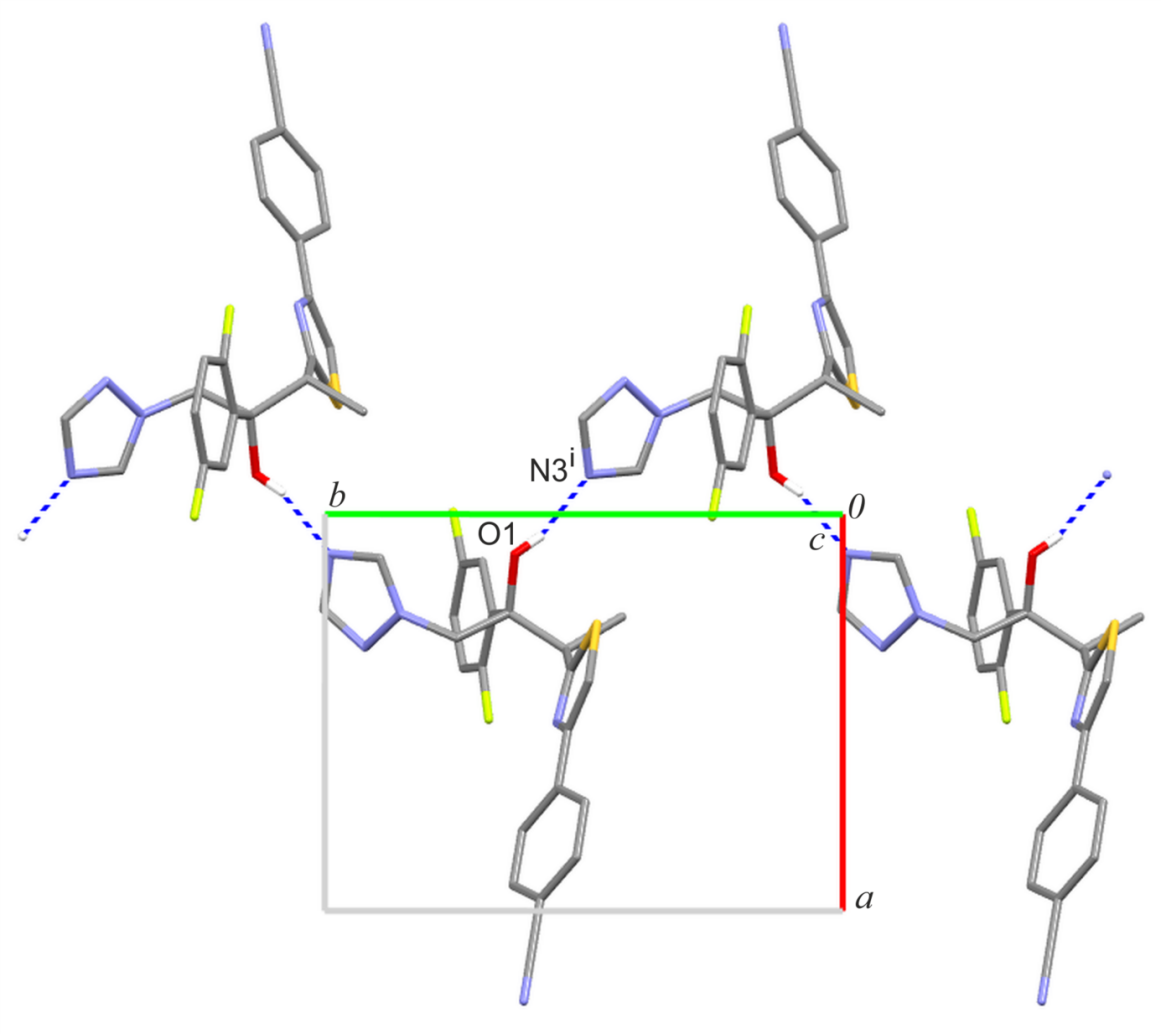
A part of the crystal structure of ISV-ortho showing a mono-periodic chain motif running along the *b-*axis direction. Hydrogen bonds are drawn as dashed lines, and for the sake of clarity, the H atoms bonded to C atoms have been omitted. Symmetry code: (i) −*x*, *y* − 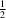, −*z* + 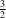.

**Figure 4:**
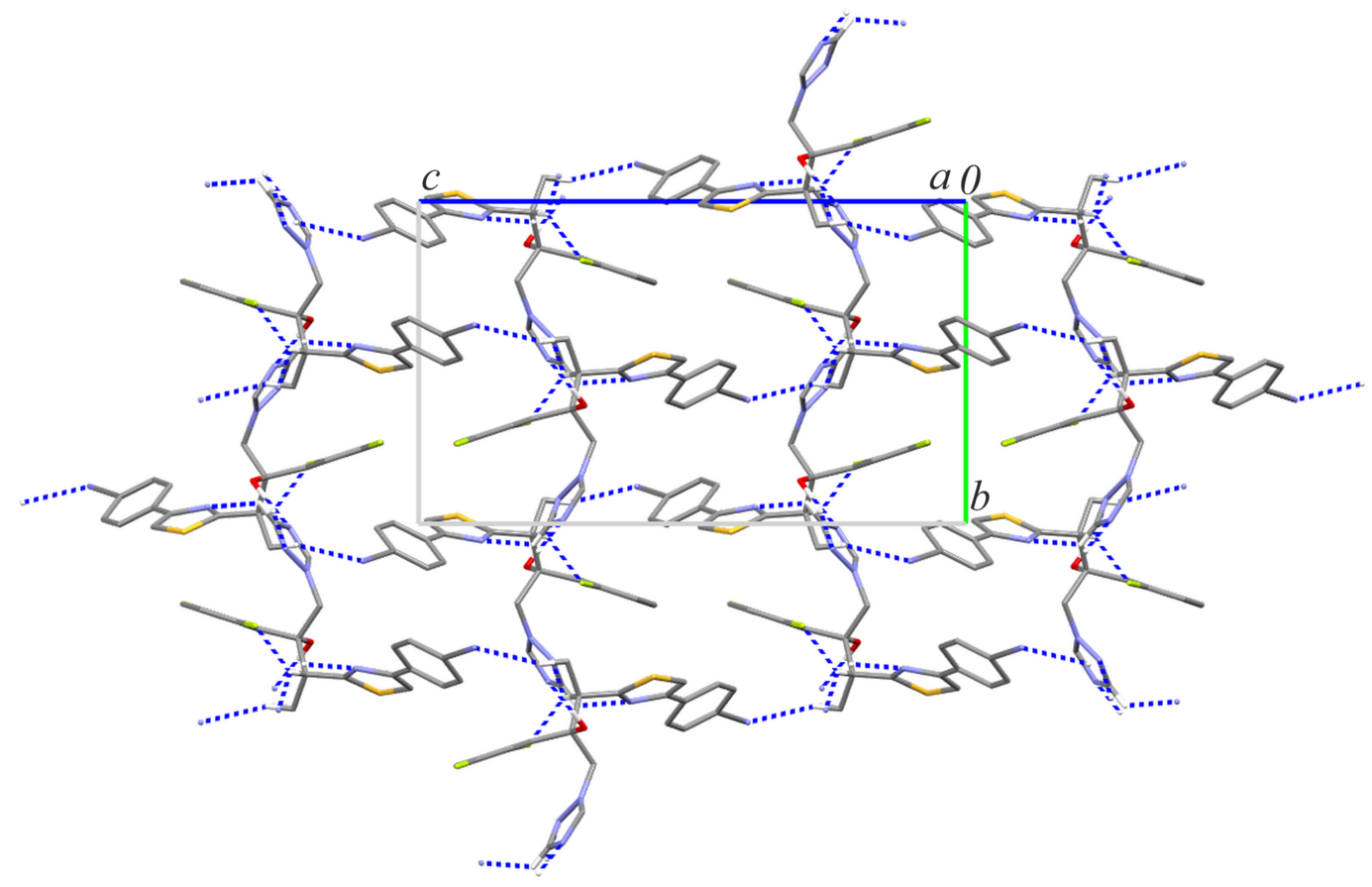
Crystal packing of ISV-ortho showing the formation of a tri-periodic supra­molecular network.

In the crystal structure of the ortho­rhom­bic form of ISV, the most important O1—H1⋯N3 hydrogen bond forms a mono-periodic chain substructure running along the crystallographic *b-*axis direction (Table 1 ▸, Fig. 3 ▸). This inter­action is the strongest with a total energy estimated as −57.80 kJ mol^−1^ and the largest contribution arising from Coulombic forces (–66.8 kJ mol^−1^; Table 2 ▸). Three additional C—H⋯N inter­actions propagate these chains into a tri-periodic supra­molecular network (Fig. 4 ▸). Among them, the mol­ecular pair connected by C4—H4⋯N4 and C11—H11⋯N2 exhibits the second highest total energy (–40.10 kJ mol^−1^) with a significant dispersive contribution (–45.3 kJ mol^−1^), likely due to supporting close contacts between the (di­fluoro)­phenyl ring and the thia­zole group. Dispersion effects are also significant for mol­ecular pairs involving contacts between triazole and benzene rings (–36.5 kJ mol^−1^). The C12—H12*B*⋯N5 inter­action leads to the formation of another sub-chain motif with a total pairwise energy of −32.0 kJ mol^−1^.

In the crystal structure of the monoclinic form of ISV, the hydroxyl group participates in an intra­molecular O—H⋯N hydrogen bond, thus only the C—H donors contribute to inter­molecular inter­actions, supported by aromatic contacts. The most significant total energies of mol­ecular pairs are summarized in Table 2 ▸. It is seen that the highest total pairwise energies are comparable in both polymorphs; however, the inter­actions responsible for them are completely different. Furthermore, the electrostatic-to-dispersive contribution ratio differs: 25:75 for the monoclinic and 42:58 for the ortho­rhom­bic form. This clearly demonstrates that the monoclinic polymorph is dominated by non-directional dispersion inter­actions, whereas the ortho­rhom­bic polymorph shows an increased contribution from Coulombic forces.

## 4. Hirshfeld surface analysis

**Figure 5:**
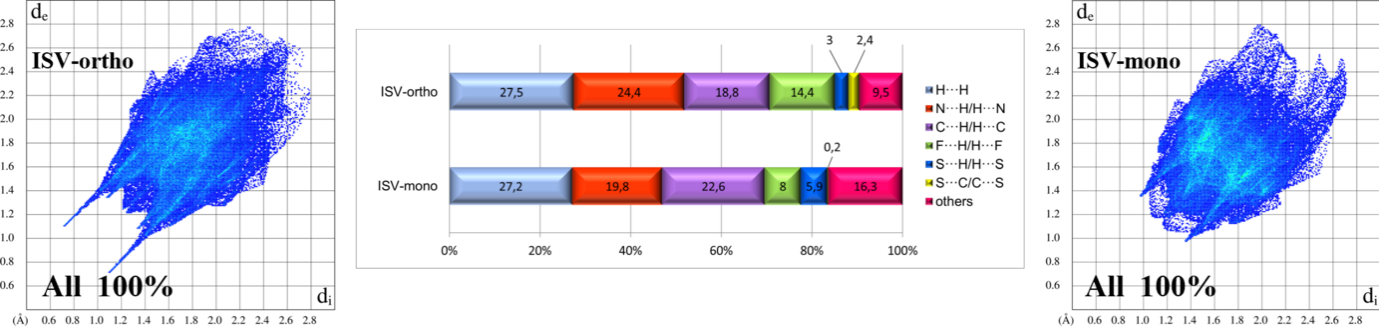
Hirshfeld surface contact contributions and two-dimensional fingerprint plots for ISV-ortho (left) and ISV-mono (right). The *d*_i_ and *d*_e_ values are the closest inter­nal and external distances (in Å) from given points on the Hirshfeld surface.

Hirshfeld surface analysis (Spackman & McKinnon, 2002 ▸; Spackman & Jayatilaka, 2009 ▸) was performed using *CrystalExplorer* (Spackman *et al.*, 2021 ▸) to visualize and qu­antify inter­molecular inter­actions in both polymorphs of isavuconazole. As shown in the breakdown diagram (Fig. 5 ▸), the major contributions to the Hirshfeld surface in both forms arise from H⋯H, N⋯H/H⋯N, and C⋯H/H⋯C contacts. The dominant share of H⋯H contacts is comparable between the two forms, whereas the proportions of N⋯H/H⋯N and C⋯H/H⋯C contacts appear complementary. Comparison of 2D fingerprint plots reveals that the main differences originate from N⋯H/H⋯N inter­actions. In the ISV-ortho form, these contacts appear as sharp, long spikes (shorter distances), while in the ISV-mono form, they are much shorter and less pronounced (longer distances). The contribution of F⋯H/H⋯F contacts is also higher in the ortho­rhom­bic form (14.4%) compared with the monoclinic one (8%). The S⋯H/H⋯S inter­actions show the opposite trend, contributing 3% in ISV-ortho *versus* 5.9% in ISV-mono, likely due to conformational differences that allow additional close contacts with the thia­zole ring in ISV-mono. In ISV-ortho, the smaller contribution of S⋯H inter­actions seem to be compensated by S⋯C contacts. Each of the other contact types contributes less than 10% in both forms.

## 5. Database survey

A search of the Cambridge Structural Database (CSD version 5.46, November 2024, Groom *et al.*, 2016 ▸) revealed four structures of isavuconazole (Voronin *et al.*, 2021 ▸): an anhydrous monoclinic form (GALJUC), a monohydrate form (GALJIQ), and two salts with phospho­ric acid (GALJOW) and *p*-toluene­sulfonic acid (GALJEM).

## 6. Synthesis and crystallization

The isavuconazole (purity 98%) used in this study was purchased from BLD Pharmatech GmbH (Germany). A pure crystalline form of isavuconazole was obtained unexpectedly from cocrystallization of the drug with pyrazinedi­carb­oxy­lic acid; all substances (0.05 mmol) were used with a fixed stoichiometric ratio of 1:1, dissolved in ethanol (3 ml EtOH) and the mixture was heated to 346 K.

## 7. Refinement

Crystal data, data collection and structure refinement details are summarized in Table 3 ▸. All hydrogen atoms bonded to carbon atoms were placed geometrically and refined as riding, with *U*_iso_(H) = 1.2 *U*_eq_(C) for the methyl­ene, methine and aromatic groups or *U*_iso_(H) = 1.5 *U*_eq_(C) for the methyl group. The hydrogen atom of the hydroxyl group was found in a difference-Fourier map.

## 8. Pairwise model energies and their energy frameworks

Pairwise model energies (Turner *et al.*, 2014 ▸) were estimated and visualized (Turner *et al.*, 2015 ▸; Mackenzie *et al.*, 2017 ▸) between mol­ecules within a cluster with a radius of 3.8 Å, using *CrystalExplorer* software (Spackman *et al.*, 2021 ▸). The computational approach uses a B3LYP/6-31G(d,p) mol­ecular wave function calculated for the respective mol­ecular arrangement in the crystal. The total inter­action energy between any nearest-neighbour mol­ecular pairs was estimated in terms of four components: electrostatic, polarization, dispersion and exchange–repulsion, with scale factors (*k*) of 1.057, 0.740, 0.871 and 0.618, respectively.

## Supplementary Material

Crystal structure: contains datablock(s) I, global. DOI: 10.1107/S2056989025008886/vm2317sup1.cif

Structure factors: contains datablock(s) I. DOI: 10.1107/S2056989025008886/vm2317Isup2.hkl

Supporting information file. DOI: 10.1107/S2056989025008886/vm2317Isup3.cml

CCDC reference: 2495049

Additional supporting information:  crystallographic information; 3D view; checkCIF report

## Figures and Tables

**Table 1:** Hydrogen-bond geometry (Å, °)

*D*—H⋯*A*	*D*—H	H⋯*A*	*D*⋯*A*	*D*—H⋯*A*
O1—H1⋯N3^i^	0.83 (2)	1.99 (3)	2.7889 (16)	164 (3)
C4—H4⋯N4^ii^	0.95	2.65	3.277 (2)	124
C11—H11⋯F1	1.00	2.24	2.9815 (17)	130
C11—H11⋯N2^iii^	1.00	2.51	3.317 (2)	138
C12—H12*B*⋯N5^iv^	0.98	2.53	3.403 (2)	149

Symmetry codes: (i) 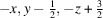; (ii) 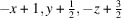; (iii) 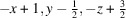; (iv) 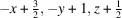.

**Table 2:** Inter­action energies (kJ mol^−1^) for the cluster of mol­ecules with a radius of 3.8 Å for ISV-ortho and ISV-mono *N* is the number of mol­ecular pairs. *R* is the distance (Å) between mol­ecular centroids. *E*_tot_ is the total energy and *E*_ele_ is the electrostatic (*k* = 1.057), *E*_pol_ is the polarization (*k* = 0.740), *E*_dis_ is the dispersion (*k* = 0.871) and *E*_rep_ is the repulsion (*k* = 0.618) component.

*N*	*R*	*kE*_ele_	*kE*_pol_	*kE*_dis_	*kE*_rep_	*E*_tot_
ISV-ortho						
2	10.15	−66.8	−13.8	−24.8	47.7	−57.8
2	15.18	−3.8	−1.0	−2.4	0.0	−7.1
2	8.95	−8.0	−1.6	−18.6	10.3	−18.0
2	11.06	−1.1	−1.3	−16.4	7.5	−11.2
2	14.04	−3.2	−0.4	−5.0	0.0	−8.5
2	8.38	−13.4	−2.4	−36.5	16.1	−36.3
2	6.79	−15.1	−3.3	−45.3	23.7	−40.1
2	12.06	−11.2	−2.4	−18.4	0.0	−32.0
2	11.70	−1.3	−0.1	−1.7	0.0	−3.0
ISV-mono						
2	9.75	2.1	−2.1	−34.3	14.4	−19.9
2	5.83	−26.1	−5.7	−70.9	42.8	−59.9
2	8.98	−9.6	−2.4	−26.1	9.0	−29.3
2	11.96	−4.5	−0.7	−5.9	2.3	−8.9
2	10.73	−2.0	−1.0	−13.3	5.1	−11.4
2	9.6	−23.1	−4.1	−24.0	19.6	−31.8
2	12.33	−0.7	−3.4	−17.9	0.0	−22.0
2	14.74	−2.2	−1.0	−2.9	0.0	−6.1

**Table 3:** Experimental details

Crystal data	
Chemical formula	C_22_H_17_F_2_N_5_OS
*M*_r_	437.46
Crystal system, space group	Orthorhombic, *P*2_1_2_1_2_1_
Temperature (K)	100
*a*, *b*, *c* (Å)	8.9485 (1), 11.6975 (1), 19.8730 (1)
*V* (Å^3^)	2080.21 (3)
*Z*	4
Radiation type	Cu *K*α
μ (mm^−1^)	1.75
Crystal size (mm)	0.22 × 0.20 × 0.05

Data collection	
Diffractometer	Rigaku XtaLAB Synergy, Dualflex, HyPix
Absorption correction	Gaussian (*CrysAlis PRO*; Rigaku OD, 2024 ▸)
*T*_min_, *T*_max_	0.228, 1.000
No. of measured, independent and observed [*I* > 2σ(*I*)] reflections	32125, 4184, 4132
*R*_int_	0.028
(sin θ/λ)_max_ (Å^−1^)	0.633

Refinement	
*R*[*F*^2^ > 2σ(*F*^2^)], *wR*(*F*^2^), *S*	0.021, 0.055, 1.03
No. of reflections	4184
No. of parameters	285
H-atom treatment	H atoms treated by a mixture of independent and constrained refinement
Δρ_max_, Δρ_min_ (e Å^−3^)	0.21, −0.19
Absolute structure	Flack *x* determined using 1711 quotients [(*I*^+^)−(*I*^−^)]/[(*I*^+^)+(*I*^−^)] (Parsons *et al.*, 2013 ▸)
Absolute structure parameter	0.002 (4)

Computer programs: *CrysAlis PRO* (Rigaku OD, 2024 ▸), *SHELXT* (Sheldrick, 2015*a* ▸), *SHELXL2019/3* (Sheldrick, 2015*b* ▸), *Mercury* (Macrae *et al.*, 2020 ▸), *PLATON* (Spek, 2020 ▸) and *publCIF* (Westrip, 2010 ▸).

## supplementary crystallographic information

### 4-{2-[(2R,3R)-3-(2,5-Difluorophenyl)-3-hydroxy-4-(1,2,4-triazol-1-yl)butan-2-yl]-1,3-thiazol-4-yl}benzonitrile Crystal data

**Table:**

C_22_H_17_F_2_N_5_OS	*D*_x_ = 1.397 Mg m^−^^3^
*M**_r_* = 437.46	Cu *K*α radiation, λ = 1.54184 Å
Orthorhombic, *P*2_1_2_1_2_1_	Cell parameters from 28179 reflections
*a* = 8.9485 (1) Å	θ = 4.4–77.0°
*b* = 11.6975 (1) Å	µ = 1.75 mm^−^^1^
*c* = 19.8730 (1) Å	*T* = 100 K
*V* = 2080.21 (3) Å^3^	Plate, colourless
*Z* = 4	0.22 × 0.20 × 0.05 mm
*F*(000) = 904	

### 4-{2-[(2R,3R)-3-(2,5-Difluorophenyl)-3-hydroxy-4-(1,2,4-triazol-1-yl)butan-2-yl]-1,3-thiazol-4-yl}benzonitrile Data collection

**Table:**

Rigaku XtaLAB Synergy, Dualflex, HyPix diffractometer	4184 independent reflections
Radiation source: micro-focus sealed X-ray tube, PhotonJet (Cu) X-ray Source	4132 reflections with *I* > 2σ(*I*)
Mirror monochromator	*R*_int_ = 0.028
Detector resolution: 10.0000 pixels mm^-1^	θ_max_ = 77.3°, θ_min_ = 4.4°
ω scans	*h* = −10→10
Absorption correction: gaussian (CrysAlisPro; Rigaku OD, 2024)	*k* = −11→14
*T*_min_ = 0.228, *T*_max_ = 1.000	*l* = −24→25
32125 measured reflections	

### 4-{2-[(2R,3R)-3-(2,5-Difluorophenyl)-3-hydroxy-4-(1,2,4-triazol-1-yl)butan-2-yl]-1,3-thiazol-4-yl}benzonitrile Refinement

**Table:**

Refinement on *F*^2^	Hydrogen site location: mixed
Least-squares matrix: full	H atoms treated by a mixture of independent and constrained refinement
*R*[*F*^2^ > 2σ(*F*^2^)] = 0.021	*w* = 1/[σ^2^(*F*_o_^2^) + (0.030*P*)^2^ + 0.3775*P*] where *P* = (*F*_o_^2^ + 2*F*_c_^2^)/3
*wR*(*F*^2^) = 0.055	(Δ/σ)_max_ = 0.001
*S* = 1.03	Δρ_max_ = 0.21 e Å^−^^3^
4184 reflections	Δρ_min_ = −0.19 e Å^−^^3^
285 parameters	Absolute structure: Flack *x* determined using 1711 quotients [(*I*^+^)-(*I*^-^)]/[(*I*^+^)+(*I*^-^)] (Parsons *et al.*, 2013)
0 restraints	Absolute structure parameter: 0.002 (4)
Primary atom site location: structure-invariant direct methods	

### 4-{2-[(2R,3R)-3-(2,5-Difluorophenyl)-3-hydroxy-4-(1,2,4-triazol-1-yl)butan-2-yl]-1,3-thiazol-4-yl}benzonitrile Special details

**Table:**

Geometry. All esds (except the esd in the dihedral angle between two l.s. planes) are estimated using the full covariance matrix. The cell esds are taken into account individually in the estimation of esds in distances, angles and torsion angles; correlations between esds in cell parameters are only used when they are defined by crystal symmetry. An approximate (isotropic) treatment of cell esds is used for estimating esds involving l.s. planes.

### 4-{2-[(2R,3R)-3-(2,5-Difluorophenyl)-3-hydroxy-4-(1,2,4-triazol-1-yl)butan-2-yl]-1,3-thiazol-4-yl}benzonitrile Fractional atomic coordinates and isotropic or equivalent isotropic displacement parameters (Å^2^)

**Table:**

	*x*	*y*	*z*	*U*_iso_*/*U*_eq_	
S1	0.27613 (4)	0.47460 (4)	0.57074 (2)	0.03016 (10)	
F1	0.51741 (10)	0.68366 (8)	0.79865 (5)	0.0253 (2)	
F2	−0.00623 (12)	0.75217 (9)	0.92935 (5)	0.0362 (2)	
O1	0.10049 (12)	0.63322 (9)	0.69664 (5)	0.0192 (2)	
N1	0.25015 (13)	0.85359 (10)	0.70417 (6)	0.0178 (2)	
N2	0.33567 (15)	0.92245 (11)	0.74385 (7)	0.0219 (3)	
N3	0.09947 (14)	0.98951 (11)	0.73679 (6)	0.0224 (3)	
N4	0.52605 (14)	0.55185 (11)	0.61439 (6)	0.0208 (3)	
N5	1.22751 (19)	0.61588 (15)	0.39819 (8)	0.0391 (4)	
C1	0.24879 (16)	0.64444 (12)	0.72044 (7)	0.0174 (3)	
C2	0.31189 (16)	0.74593 (12)	0.67995 (7)	0.0189 (3)	
H2A	0.422088	0.747449	0.684257	0.023*	
H2B	0.286951	0.736175	0.631761	0.023*	
C3	0.11079 (17)	0.89399 (12)	0.70097 (8)	0.0202 (3)	
H3	0.031324	0.859480	0.676635	0.024*	
C4	0.24030 (17)	1.00243 (13)	0.76200 (8)	0.0228 (3)	
H4	0.267423	1.064314	0.790447	0.027*	
C5	0.25190 (16)	0.67557 (11)	0.79592 (7)	0.0183 (3)	
C6	0.38393 (17)	0.69634 (13)	0.83090 (8)	0.0210 (3)	
C7	0.3890 (2)	0.73257 (13)	0.89697 (8)	0.0258 (3)	
H7	0.482322	0.744423	0.918691	0.031*	
C8	0.2569 (2)	0.75139 (13)	0.93113 (7)	0.0284 (3)	
H8	0.256705	0.776037	0.976678	0.034*	
C9	0.12582 (19)	0.73321 (14)	0.89692 (8)	0.0255 (3)	
C10	0.11984 (17)	0.69603 (13)	0.83093 (8)	0.0210 (3)	
H10	0.026052	0.684490	0.809584	0.025*	
C11	0.34188 (16)	0.53393 (12)	0.70649 (7)	0.0196 (3)	
H11	0.434223	0.539147	0.734658	0.023*	
C12	0.25850 (19)	0.42638 (13)	0.72888 (8)	0.0257 (3)	
H12A	0.169462	0.415987	0.700824	0.038*	
H12B	0.228474	0.434422	0.776063	0.038*	
H12C	0.324116	0.359800	0.724093	0.038*	
C13	0.39205 (17)	0.52523 (13)	0.63439 (7)	0.0208 (3)	
C14	0.42004 (19)	0.49034 (15)	0.51479 (8)	0.0292 (3)	
H14	0.414034	0.473052	0.468164	0.035*	
C15	0.54393 (18)	0.53089 (13)	0.54615 (7)	0.0234 (3)	
C16	0.69074 (18)	0.54990 (13)	0.51454 (8)	0.0244 (3)	
C17	0.7979 (2)	0.61881 (15)	0.54546 (8)	0.0306 (4)	
H17	0.775707	0.654112	0.587329	0.037*	
C18	0.9363 (2)	0.63651 (17)	0.51599 (9)	0.0341 (4)	
H18	1.008092	0.684119	0.537319	0.041*	
C19	0.9696 (2)	0.58388 (15)	0.45471 (8)	0.0291 (4)	
C20	0.86455 (19)	0.51375 (14)	0.42361 (8)	0.0275 (3)	
H20	0.887849	0.477219	0.382228	0.033*	
C21	0.7261 (2)	0.49738 (13)	0.45314 (7)	0.0262 (3)	
H21	0.654342	0.450069	0.431590	0.031*	
C22	1.1127 (2)	0.60168 (16)	0.42323 (9)	0.0326 (4)	
H1	0.056 (3)	0.584 (2)	0.7187 (12)	0.040 (6)*	

### 4-{2-[(2R,3R)-3-(2,5-Difluorophenyl)-3-hydroxy-4-(1,2,4-triazol-1-yl)butan-2-yl]-1,3-thiazol-4-yl}benzonitrile Atomic displacement parameters (Å^2^)

**Table:**

	*U*^11^	*U*^22^	*U*^33^	*U*^12^	*U*^13^	*U*^23^
S1	0.02487 (18)	0.0387 (2)	0.02694 (18)	0.00058 (17)	−0.00362 (15)	−0.01020 (16)
F1	0.0180 (4)	0.0266 (5)	0.0312 (5)	−0.0017 (4)	−0.0013 (4)	−0.0015 (4)
F2	0.0370 (5)	0.0408 (5)	0.0306 (5)	0.0009 (5)	0.0150 (4)	−0.0053 (4)
O1	0.0168 (5)	0.0170 (5)	0.0238 (5)	−0.0015 (4)	−0.0019 (4)	0.0011 (4)
N1	0.0182 (6)	0.0153 (5)	0.0198 (5)	−0.0005 (5)	0.0003 (5)	0.0009 (4)
N2	0.0202 (6)	0.0190 (6)	0.0266 (6)	−0.0021 (5)	−0.0020 (5)	−0.0023 (5)
N3	0.0204 (6)	0.0182 (6)	0.0286 (6)	0.0016 (5)	−0.0009 (5)	−0.0011 (5)
N4	0.0245 (6)	0.0174 (6)	0.0204 (6)	0.0047 (5)	0.0004 (5)	−0.0008 (5)
N5	0.0384 (9)	0.0498 (9)	0.0293 (7)	0.0012 (8)	0.0074 (7)	0.0022 (7)
C1	0.0165 (7)	0.0157 (6)	0.0199 (6)	0.0003 (5)	−0.0001 (5)	−0.0004 (5)
C2	0.0191 (7)	0.0163 (7)	0.0214 (6)	0.0016 (5)	0.0021 (5)	0.0002 (6)
C3	0.0188 (7)	0.0179 (7)	0.0238 (7)	0.0000 (6)	−0.0009 (6)	0.0007 (6)
C4	0.0222 (7)	0.0184 (7)	0.0279 (7)	−0.0009 (6)	−0.0022 (6)	−0.0028 (5)
C5	0.0212 (7)	0.0134 (6)	0.0205 (6)	−0.0005 (5)	0.0000 (6)	0.0013 (5)
C6	0.0215 (7)	0.0166 (7)	0.0249 (7)	−0.0003 (6)	0.0010 (6)	0.0010 (6)
C7	0.0310 (8)	0.0207 (8)	0.0257 (7)	−0.0034 (7)	−0.0069 (7)	0.0000 (6)
C8	0.0422 (9)	0.0229 (7)	0.0202 (6)	−0.0021 (7)	0.0006 (7)	−0.0019 (6)
C9	0.0298 (8)	0.0217 (8)	0.0251 (7)	0.0008 (6)	0.0085 (6)	−0.0002 (6)
C10	0.0219 (7)	0.0171 (7)	0.0240 (7)	−0.0009 (6)	0.0017 (6)	0.0007 (6)
C11	0.0197 (6)	0.0172 (7)	0.0218 (7)	0.0022 (6)	−0.0012 (5)	−0.0008 (6)
C12	0.0277 (8)	0.0166 (7)	0.0327 (8)	0.0036 (6)	0.0021 (7)	0.0026 (6)
C13	0.0218 (7)	0.0166 (7)	0.0239 (7)	0.0039 (6)	−0.0022 (6)	−0.0030 (6)
C14	0.0313 (8)	0.0332 (9)	0.0231 (7)	0.0064 (7)	−0.0030 (6)	−0.0064 (7)
C15	0.0303 (8)	0.0197 (7)	0.0203 (7)	0.0068 (6)	−0.0001 (6)	−0.0016 (6)
C16	0.0307 (8)	0.0226 (7)	0.0200 (7)	0.0077 (6)	0.0011 (6)	0.0023 (6)
C17	0.0348 (9)	0.0330 (9)	0.0240 (7)	0.0021 (7)	0.0039 (7)	−0.0056 (7)
C18	0.0341 (9)	0.0384 (10)	0.0297 (8)	−0.0004 (8)	0.0037 (7)	−0.0041 (7)
C19	0.0337 (9)	0.0300 (8)	0.0237 (7)	0.0079 (7)	0.0050 (7)	0.0049 (6)
C20	0.0382 (9)	0.0248 (7)	0.0195 (7)	0.0110 (7)	0.0023 (6)	0.0024 (6)
C21	0.0358 (8)	0.0223 (7)	0.0203 (6)	0.0062 (7)	−0.0007 (6)	0.0007 (5)
C22	0.0378 (9)	0.0362 (9)	0.0238 (7)	0.0083 (8)	0.0034 (7)	0.0019 (7)

### 4-{2-[(2R,3R)-3-(2,5-Difluorophenyl)-3-hydroxy-4-(1,2,4-triazol-1-yl)butan-2-yl]-1,3-thiazol-4-yl}benzonitrile Geometric parameters (Å, º)

**Table:**

S1—C14	1.7112 (17)	C7—H7	0.9500
S1—C13	1.7398 (15)	C8—C9	1.372 (2)
F1—C6	1.3636 (18)	C8—H8	0.9500
F2—C9	1.3642 (18)	C9—C10	1.383 (2)
O1—C1	1.4149 (17)	C10—H10	0.9500
O1—H1	0.83 (3)	C11—C13	1.505 (2)
N1—C3	1.335 (2)	C11—C12	1.529 (2)
N1—N2	1.3625 (18)	C11—H11	1.0000
N1—C2	1.4570 (18)	C12—H12A	0.9800
N2—C4	1.317 (2)	C12—H12B	0.9800
N3—C3	1.329 (2)	C12—H12C	0.9800
N3—C4	1.3645 (19)	C14—C15	1.357 (2)
N4—C13	1.301 (2)	C14—H14	0.9500
N4—C15	1.3875 (19)	C15—C16	1.473 (2)
N5—C22	1.154 (2)	C16—C17	1.396 (2)
C1—C2	1.5412 (19)	C16—C21	1.402 (2)
C1—C5	1.5438 (19)	C17—C18	1.385 (3)
C1—C11	1.5627 (19)	C17—H17	0.9500
C2—H2A	0.9900	C18—C19	1.397 (2)
C2—H2B	0.9900	C18—H18	0.9500
C3—H3	0.9500	C19—C20	1.392 (3)
C4—H4	0.9500	C19—C22	1.441 (2)
C5—C10	1.392 (2)	C20—C21	1.384 (2)
C5—C6	1.392 (2)	C20—H20	0.9500
C6—C7	1.380 (2)	C21—H21	0.9500
C7—C8	1.381 (2)		
			
C14—S1—C13	89.26 (7)	C9—C10—H10	120.2
C1—O1—H1	110.0 (16)	C5—C10—H10	120.2
C3—N1—N2	110.06 (12)	C13—C11—C12	111.54 (12)
C3—N1—C2	130.12 (12)	C13—C11—C1	112.57 (12)
N2—N1—C2	119.29 (12)	C12—C11—C1	111.65 (11)
C4—N2—N1	102.39 (12)	C13—C11—H11	106.9
C3—N3—C4	102.67 (12)	C12—C11—H11	106.9
C13—N4—C15	111.26 (13)	C1—C11—H11	106.9
O1—C1—C2	103.92 (11)	C11—C12—H12A	109.5
O1—C1—C5	111.32 (11)	C11—C12—H12B	109.5
C2—C1—C5	108.60 (11)	H12A—C12—H12B	109.5
O1—C1—C11	111.34 (11)	C11—C12—H12C	109.5
C2—C1—C11	110.44 (11)	H12A—C12—H12C	109.5
C5—C1—C11	110.97 (11)	H12B—C12—H12C	109.5
N1—C2—C1	110.76 (11)	N4—C13—C11	123.34 (13)
N1—C2—H2A	109.5	N4—C13—S1	114.13 (11)
C1—C2—H2A	109.5	C11—C13—S1	122.51 (11)
N1—C2—H2B	109.5	C15—C14—S1	110.73 (11)
C1—C2—H2B	109.5	C15—C14—H14	124.6
H2A—C2—H2B	108.1	S1—C14—H14	124.6
N3—C3—N1	110.08 (13)	C14—C15—N4	114.60 (14)
N3—C3—H3	125.0	C14—C15—C16	125.84 (14)
N1—C3—H3	125.0	N4—C15—C16	119.53 (14)
N2—C4—N3	114.79 (13)	C17—C16—C21	118.74 (15)
N2—C4—H4	122.6	C17—C16—C15	120.84 (14)
N3—C4—H4	122.6	C21—C16—C15	120.41 (15)
C10—C5—C6	116.15 (13)	C18—C17—C16	120.97 (15)
C10—C5—C1	120.72 (13)	C18—C17—H17	119.5
C6—C5—C1	122.80 (13)	C16—C17—H17	119.5
F1—C6—C7	116.84 (14)	C17—C18—C19	119.56 (17)
F1—C6—C5	119.31 (13)	C17—C18—H18	120.2
C7—C6—C5	123.82 (15)	C19—C18—H18	120.2
C6—C7—C8	119.22 (15)	C20—C19—C18	120.19 (16)
C6—C7—H7	120.4	C20—C19—C22	119.51 (15)
C8—C7—H7	120.4	C18—C19—C22	120.30 (17)
C9—C8—C7	117.61 (13)	C21—C20—C19	119.83 (15)
C9—C8—H8	121.2	C21—C20—H20	120.1
C7—C8—H8	121.2	C19—C20—H20	120.1
F2—C9—C8	118.75 (14)	C20—C21—C16	120.70 (15)
F2—C9—C10	117.76 (15)	C20—C21—H21	119.7
C8—C9—C10	123.49 (15)	C16—C21—H21	119.7
C9—C10—C5	119.69 (14)	N5—C22—C19	179.8 (2)
			
C3—N1—N2—C4	−0.52 (15)	C2—C1—C11—C13	−37.30 (16)
C2—N1—N2—C4	−172.96 (12)	C5—C1—C11—C13	−157.78 (12)
C3—N1—C2—C1	−67.54 (18)	O1—C1—C11—C12	−48.73 (15)
N2—N1—C2—C1	103.15 (14)	C2—C1—C11—C12	−163.64 (12)
O1—C1—C2—N1	73.99 (13)	C5—C1—C11—C12	75.87 (15)
C5—C1—C2—N1	−44.61 (15)	C15—N4—C13—C11	177.28 (13)
C11—C1—C2—N1	−166.50 (11)	C15—N4—C13—S1	−1.04 (16)
C4—N3—C3—N1	−0.67 (16)	C12—C11—C13—N4	−133.40 (15)
N2—N1—C3—N3	0.79 (16)	C1—C11—C13—N4	100.19 (16)
C2—N1—C3—N3	172.15 (13)	C12—C11—C13—S1	44.79 (17)
N1—N2—C4—N3	0.10 (17)	C1—C11—C13—S1	−81.62 (15)
C3—N3—C4—N2	0.35 (18)	C14—S1—C13—N4	0.40 (13)
O1—C1—C5—C10	−3.97 (18)	C14—S1—C13—C11	−177.93 (13)
C2—C1—C5—C10	109.85 (14)	C13—S1—C14—C15	0.37 (14)
C11—C1—C5—C10	−128.58 (14)	S1—C14—C15—N4	−1.05 (19)
O1—C1—C5—C6	−177.17 (12)	S1—C14—C15—C16	177.01 (12)
C2—C1—C5—C6	−63.35 (17)	C13—N4—C15—C14	1.36 (19)
C11—C1—C5—C6	58.23 (17)	C13—N4—C15—C16	−176.83 (13)
C10—C5—C6—F1	−176.72 (13)	C14—C15—C16—C17	162.90 (17)
C1—C5—C6—F1	−3.2 (2)	N4—C15—C16—C17	−19.1 (2)
C10—C5—C6—C7	1.5 (2)	C14—C15—C16—C21	−18.1 (2)
C1—C5—C6—C7	174.96 (14)	N4—C15—C16—C21	159.86 (14)
F1—C6—C7—C8	177.32 (13)	C21—C16—C17—C18	0.7 (2)
C5—C6—C7—C8	−0.9 (2)	C15—C16—C17—C18	179.76 (16)
C6—C7—C8—C9	−0.3 (2)	C16—C17—C18—C19	−0.5 (3)
C7—C8—C9—F2	−179.42 (14)	C17—C18—C19—C20	−0.3 (3)
C7—C8—C9—C10	0.8 (2)	C17—C18—C19—C22	179.77 (17)
F2—C9—C10—C5	−179.98 (13)	C18—C19—C20—C21	0.8 (2)
C8—C9—C10—C5	−0.2 (2)	C22—C19—C20—C21	−179.20 (15)
C6—C5—C10—C9	−0.9 (2)	C19—C20—C21—C16	−0.6 (2)
C1—C5—C10—C9	−174.52 (13)	C17—C16—C21—C20	−0.2 (2)
O1—C1—C11—C13	77.62 (14)	C15—C16—C21—C20	−179.18 (14)

### 4-{2-[(2R,3R)-3-(2,5-Difluorophenyl)-3-hydroxy-4-(1,2,4-triazol-1-yl)butan-2-yl]-1,3-thiazol-4-yl}benzonitrile Hydrogen-bond geometry (Å, º)

**Table:**

*D*—H···*A*	*D*—H	H···*A*	*D*···*A*	*D*—H···*A*
O1—H1···N3^i^	0.83 (2)	1.99 (3)	2.7889 (16)	164 (3)
C4—H4···N4^ii^	0.95	2.65	3.277 (2)	124
C11—H11···F1	1.00	2.24	2.9815 (17)	130
C11—H11···N2^iii^	1.00	2.51	3.317 (2)	138
C12—H12*B*···N5^iv^	0.98	2.53	3.403 (2)	149

Symmetry codes: (i) −*x*, *y*−1/2, −*z*+3/2; (ii) −*x*+1, *y*+1/2, −*z*+3/2; (iii) −*x*+1, *y*−1/2, −*z*+3/2; (iv) −*x*+3/2, −*y*+1, *z*+1/2.
